# Diagnosis of tuberculosis in an Indian population by an indirect ELISA protocol based on detection of Antigen 85 complex: a prospective cohort study

**DOI:** 10.1186/1471-2334-7-74

**Published:** 2007-07-10

**Authors:** Rajpal S Kashyap, Anju N Rajan, Sonali S Ramteke, Vijay S Agrawal, Sanjivani S Kelkar, Hemant J Purohit, Girdhar M Taori, Hatim F Daginawala

**Affiliations:** 1 Biochemistry Research Laboratory, Central India Institute of Medical Sciences, Nagpur-10, India; 2 Environmental Genomics Unit, National Environmental Engineering Research Institute, Nehru Marg, Nagpur-440020, India

## Abstract

**Background:**

Diagnosis of tuberculosis (TB) remains problematic despite many new advanced diagnostic methods. A reliable and rapid diagnostic test, which could be performed in any standard pathology laboratory, would help to obtain definitive early diagnoses of TB. In the present study we describe a prospective evaluation for demonstrating Antigen (Ag) 85 complex in the sera from TB patients.

**Methods:**

Indirect ELISA, employing monoclonal antibodies (mAb) against the purified Ag 85 complex, was used to demonstrate Ag 85 complex in sera from TB patients. Serum samples were obtained from 197 different groups of patients: confirmed TB {n = 24}, clinically diagnosed TB {n = 104}, disease controls {n = 49} and healthy controls {n = 20}. Receiver operating curve (ROC) was used to calculate the cut off value and comparison between TB and non-TB groups were done by the chi-square test.

**Results:**

The indirect ELISA method, using an mAb against Ag 85 complex, yielded 82% sensitivity (95% confidence interval [CI] 67 to 93%) and 86% specificity (95% CI, 57 to 98%) for the diagnosis of TB. The serum positivities for Ag 85 complex in cases of confirmed and clinically diagnosed TB patients were 96% (23/24) and 79% (82/104) respectively, while the positivity for patients in the non-tuberculosis group was 14% (10/69).

**Conclusion:**

The detection of Ag 85 complex in sera from TB patients by indirect ELISA using mAb against purified Ag 85 complex gives a reliable diagnosis and can be used to develop an immunodiagnostic assay with increased sensitivity and specificity.

## Background

One third of the global population is believed to be infected with *M. tuberculosis *bacilli (MTB) complex, the causative agent of tuberculosis (TB) [1]. The recent increase of this endemic disease is due to its occurrence in association with human immunodeficiency virus (HIV); this co-infection has aggravated the existing situation [2]. Fast and accurate diagnosis of TB is very important element in global health measures to control the disease [3]. Traditionally, diagnosis of TB rests on sputum examination and cultures for acid-fast bacilli (AFB) [4]. However, the sensitivity of the sputum smear for AFB is very poor and facilities for mycobacterial culture are often scarce in regions where tuberculosis is common; and even when they are available, culture results are frequently usually too late to affect initial management [5].

The Mantoux Tuberculin Skin Test (TST), which uses five tuberculin units of purified protein derivative, is the standard routine method for detecting *M. tuberculosis *infection. Since TST is generally used to determine asymptomatic infection, the false-negative rate cannot be calculated. A negative TST does not rule out TB disease in a child. False-positive reactions to TST are often attributed to asymptomatic infection by non-tuberculous mycobacteria in the environment. The relatively low sensitivity and specificity of TST makes the test very useful for people at high risk of TB infection or disease but undesirable for people at low risk [6,7]. The QuantiFerron TB test was recently developed to overcome some of the limitations of the TST; however, the 12 hrs time limit on whole blood processing is a major weakness in terms of its applications in a reference laboratory setting [8]. Recently Diagnostic polymerase chain reaction (PCR) using specific primers as markers for *M. tuberculosis *is not performed correctly in all clinical laboratories and shows variable sensitivity and specificity [9]. Many serological assays have been tried, but nearly all have failed to improve upon the time-honored sputum smear and culture approach [10-14].

We have previously demonstrated the presence of a 30-kD-protein antigen in cerebrospinal fluid (CSF) from confirmed and suspected tuberculous meningitis (TBM) patients [15]. This 30 kD protein band was excised from the gel, digested with trypsin and analyzed by liquid chromatography tandem mass spectrometry (LC-MS/MS) and two-dimensional polyacrylamide gel electrophoresis (2DPAGE). Together, these studies identified the components of the TBM-specific 30 kD antigen: two mycobacterial antigens, Rv 3804c and Rv1886c (Ag 85 A and B respectively, both members of Ag 85 complex), and one host-derived protein (immunoglobulin [Ig] Kappa light chain VLJ region; accession no. BAC01690.1) [16]. Concurrently, antibodies generated against the 30-kD protein were found to react with most CSF samples from TBM patients.

Ag85 complex comprises three related major secretary proteins of *M. tuberculosis*, which have been the focus of extensive research for several years: Ag85A (31 kD), Ag85B (30 kD) and Ag85C (31.5 kD). These antigens have also been demonstrated in the sputum of pulmonary TB patients [17]. Various forms of Ag 85 complex have previously been evaluated for antibody detection in extra-central nervous system TB [18-20]. In the present study, serum specimens from patients with TB were examined for the presence of *M. tuberculosis *Ag 85 complex by a sensitive and specific indirect ELISA protocol developed in our laboratory.

## Methods

### Study subjects

We prospectively selected serum samples from 128 active TB patients (81 male, 47 female; age 13–63 years) from inpatient and outpatient services at the Tuberculosis Center, Government Medical College (GMC), Nagpur and Central India Institute of Medical Sciences, Nagpur. In addition, selected patients admitted to the hospital for defined acute or chronic non-TB diseases (*n *= 49) including asthma (*n *= 10), neurological disorders (*n *= 09), respiratory symptoms (*n *= 08), gastrointestinal symptoms (*n *= 8), non-specific fever (n = 8), pneumonia (*n *= 2), bronchitis (*n *= 2), lung cancer (*n *= 1) and lung infection (*n *= 1), as well as 20 healthy volunteers (age 10–56) with no signs of clinical impairment and normal chest radiographs, were included as controls. All subjects were negative for HIV. Serum samples were obtained from almost all patients before initiation of AKT and were stored at -20°C until they were tested. In India, BCG vaccination is given within one week of the birth of child; all subjects included in the study had been vaccinated with BCG. Samples were collected from all study groups for which patient's consent was obtained.

To diagnose active TB, sputum microscopy was done on two serial sputum samples by staining with Ziehl Neelson Stain as per the guidelines of India's Revised National Tuberculosis Control Programme. However, Of the 128 patients, only 24 patients had initial positive results for AFB in sputum samples. Final cultures for *M.tuberculosis *on Lowenstein-Jensen medium obtained after 6 weeks were positive. TB was confirmed if AFB and/or culture of sputum specimens were positive for *M. tuberculosis*. When both tests were negative, the patients were diagnosed by clinical symptoms. Clinical suspicion of tuberculosis was based on minimum of 3 symptoms of the following a) Chronic cough with or without expectoration/hemoptysis/chest pain of more than 2–3 weeks or past history of TB b) Fever more than 2–3 weeks c) Progressive unexplained weight loss d) loss of appetite e) night sweats. Radiographic features supporting the clinical diagnosis considered were lung parenchymal infiltration mainly involving apical and/or mid zone, miliary shadows and pleural effusion. Along with the above mentioned clinical features any one radiological feature was considered sufficient as supportive evidence. Ultrasound examination of chest was done in suspected cases of pleural effusion, which was also utilized for diagnostic pleural tap. Sputum samples of 41 TB patients with clinical diagnosis was available but were all negative to culture/AFB staining. All patients received anti-TB drug (not covered under DOT) in absence of any other therapy. Daily dosages were given as isoniazid 300 mg, rifampicin 450 mg or 600 mg, pyrazinamide 1.5 g or 2.0 g and ethambutol 25 mg/kg for the first 2 months. In the next 2 months rifampicin 450 mg or 600 mg, isoniazid 300 mg and ethambutol were given followed by ethambutol, rifampicin 450 mg or 600 mg and isoniazid 300 mg for the next 4 months.

Cases were followed at regular interval for a period of 9 months. Improvement in all subjects was judged clinically (Improvement in cough, fever, appetite, wieight gain etc) radiographic evidence (resolution of lesion on repeat chest × ray after 3 months of standard anti-TB treatment). All subjects including clinically diagnosed patients improved clinically and responded well to anti-Koch treatment (AKT) after three months of standard anti-tuberculosis treatment. The Central India Institute of Medical Sciences Ethical Committee, Nagpur, India approved the study and all the analyses were performed double blinded.

### Specimens

Sputum specimens for ordinary examination by AFB and cultivation were obtained over three consecutive days. The sputum sample was digested and decontaminated with 2% sodium hydroxide and then processed for further investigation. Ziehl-Neelson acid fast staining was used to confirm the presence of acid-fast bacilli. Venous blood was collected from all the patients and control subjects. Blood was allowed to clot, and after centrifugation (1000 × *g*, 10 min) the serum was separated and stored at -20°C until it was used.

### Antigen and antibody

The purified Ag 85 complex and monoclonal antibodies against this complex (CS-90) were obtained from Colorado State University, USA through the TB Research Materials and Vaccine Testing Contract (NO1-AI-40091).

### Indirect ELISA protocol

Prior to patient sampling, the assay was standardized by incubating purified Ag 85 complex with the CS-90 mAb at different dilutions (1–500 ng/ml). Indirect ELISA was performed as described by Kashyap et al. [16]. Serum samples of 100 μl (1:200) from TB patients and control subjects were added to the microtiter wells and blocked with 0.5% bovine serum albumin (BSA) in phosphate buffered saline (PBS). After washing with PBS, the mAb (1:2000) was added and the plates were incubated at 37°C for 60 min. The wells were washed, and then secondary antibody (goat anti-rabbit IgG-HRP, 1:10000) was added and incubated for 60 min at 37°C. After another wash with PBS, 100 μl of TMB/H_2_O_2 _substrate was added to the wells and incubated at room temperature for about 10 min. The reaction was then stopped with 100 μl of 2.5 N H_2_SO_4_. The absorbance of each well was read at 450 nm. Samples with absorbance > 0.18 were considered positive. Each sample was tested in triplicate.

### Western blotting

Serum proteins at 30 μg/lane were separated by sodium dodecyl sulfate polyacrylamide gel electrophoresis (SDS-PAGE) [26] and transferred to polyvinyledineflouride (PVDF) membranes by electroblotting at 100 V for 3 hours. The membrane was treated with 50% v/v methanol immediately prior to and after the electroblotting. The membrane was then blocked with 0.5% BSA in PBS at 37°C for 60 min. After blocking, the membrane was washed with PBS (3 × 10 min), probed with CS-90 mAb (1:2000) and incubated at 37°C for 60 min. The membrane was then washed with PBS, followed by addition of 1:10,000 diluted affinity-purified anti-rabbit IgG conjugated to horseradish peroxidase (Genei, Bangalore, India) and incubated at 37°C for 60 min. After incubation, the membrane was washed extensively with PBS followed by addition of tetramethylbenzidine-hydrogen peroxide (TMB/H_2_O_2_), which enabled the antibody reaction to be visualized.

### Statistical analysis

The sensitivities and specificities of developed Indirect ELISA based test for diagnosis of TB and Non-TB group were calculated. The positive and negative predicative values were calculated by using different rates of TB prevalence. Receiver operating curve (ROC) was used to calculate the cut off value and comparison between TB and non-TB groups was done by the chi-square test.

## Results

**Figure 1:**
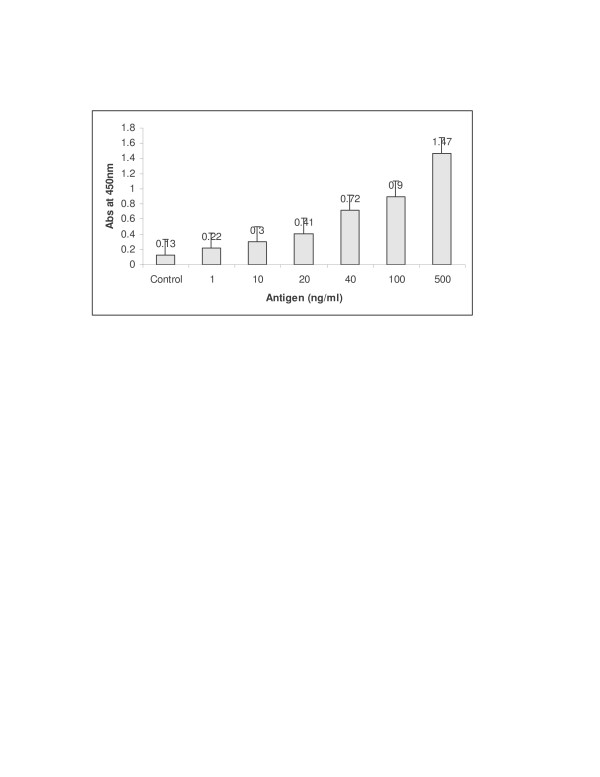
Absorbance at 450 nm with increasing concentration of Ag 85 complex in the standardization of the indirect ELISA method.

Different concentrations of Ag 85 complex antigen were titrated with the antibody and a standard curve was plotted. Figure 1 shows the increase in absorbance at 450 nm with increasing concentration of Ag 85 complex antigen during the standardization of the indirect ELISA method.

Table-1 shows the occurrence of Ag 85 complex antigen in sera from the TB and non-TB groups as determined by the indirect ELISA method along with mean absorbance (with range). Cutoff value of 0.18 was determined for ELISA test using ROC analysis. The serum positivities for Ag 85 complex antigen in cases of confirmed and clinically diagnosed TB patients were 96% (23/24) and 79% (82/104) respectively, while the positivity for patients in the non-tuberculosis group was 14% (10/69). No Ag 85 complex was detected in the healthy control group. Overall, the indirect ELISA method yielded 82% sensitivity (95% confidence interval [CI] 67 to 93%) and 86% specificity (95% CI, 57 to 98%) for the diagnosis of TB using the mAb to Ag 85 complex. The mean absorbance value of Ag 85 complex antigen in the TB patients was 0.42 ± 0.22 (range 0.03–0.92), significantly higher than in the non-TB group (0.16 ± 0.08; range 0.01–0.36; *P *< 0.001). There was a significant difference in the mean Ag 85 complex antigen activity between the confirmed TB patients (0.64 ± 0.22; range 0.22 – 0.92) and the clinically diagnosed TB patients (0.37 ± 0.19; range 0.03 – 0.92;*P *< 0.0001) (Table-1).

**Table 1:** Demonstration of Ag 85 complex in sera from tuberculosis and non-tuberculosis patients by indirect ELISA method using the mAb against purified Ag 85 complex along with mean absorbance (with range)

**Patient group**	**Positivity for Ag 85 complex **(cutoff of 0.18)	**Negativity for Ag 85 complex**	**Absorbance (Mean ± 2SD)**	**Range**
**Tuberculosis group (n = 128)**	**105 (82.03%)**	**23 (17.96%)**	**0.42 ± 0.22**	**0.03 – 0.92**
Confirmed (n = 24) (AFB & ulture positive)	23 (95.83%)	1 (4.17%)	0.64 ± 0.22	0.22 – 0.92
Clinically diagnosed (n = 104) (AFB & ulture negative)	82 (78.84%)	22 (21.16%)	0.37 ± 0.19	0.03 – 0.92

**Non-tuberculosis group (69)**	**10(14. 49%)**	**59 (85. 51%)**	**0.16 ± 0.08**	**0.01 – 0.36**
Disease Control(n = 49)	10 (20.40%)	39 (79.59%)	0.17 ± 0.09	0.01 – 0.36
Healthy Control(n = 20)	0 (0%)	20 (100%)	0.13 ± 0.05	0.02 – 0.21

The data are expressed as mean ± SD.

**Figure 2:**
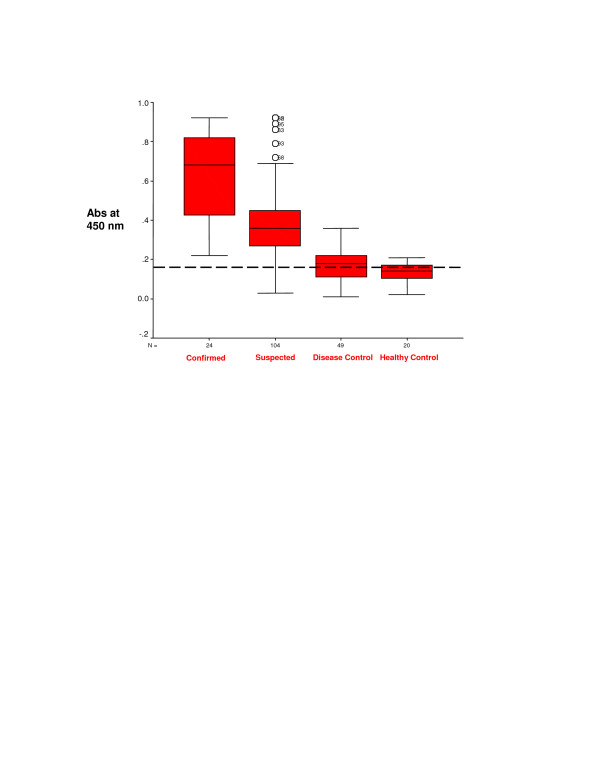
Box plot demonstrating Ag 85 complex in sera from confirmed and clinically diagnosed TB patients, non-TB disease patients and healthy control group. The box plot shows the 5^th ^and 95 th percentiles (bars), 75 th and 25^th ^percentiles (boxes) and median (bars in boxes). N -numbers of individual in each group. (---- cut-off value).

**Figure 3:**
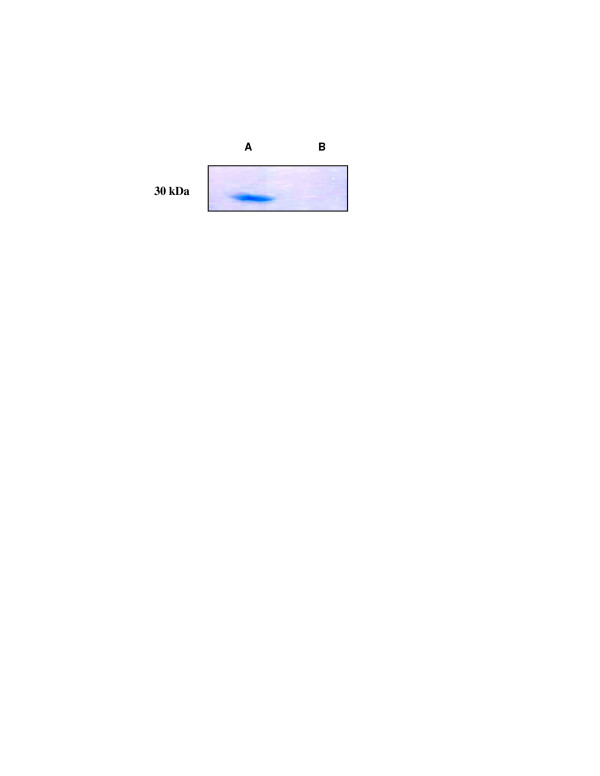
Immunoblot analysis using specific antibodies against Ag85 in serum samples from TB (lane A) and non-TB patients (lane B). Note the presence of Ag 85 complex (30 kD) only in TB patient serum.

Box plots of the Ag 85 complex activity in sera from confirmed and clinically diagnosed TB patients, non-TB disease patients and healthy control group with cut off value are shown in figure 2 together with the 90^th ^percentile range, 75 th and 25 th percentiles. The serum samples from the TB and non-TB patients were separated by one-dimensional electrophoresis and blotted on to PVDF membranes. Figure 3 depicts immunoblotting with specific rabbit antibodies against Ag 85 complex antigen. The serum of a TB patient (lane 1) shows reactivity for Ag 85 complex antigen, which was absent in case of a non-TB patient (lane 2).

## Discussion

Various immunoassays for detecting antigen or antibody in serum samples with different sensitivities and specificities have been developed. However, despite extensive work on the diagnosis of TB, only few diagnostic tests are available [21-24]. We previously isolated a 30 kD protein from CSF of TBM patients and characterized it using LC-MS/MS analysis and N-terminal sequencing. These studies demonstrated that the 30 kD protein contains Rv3804c (Ag 85A) and Rv1886 c (Ag 85 B), both components of the Ag 85 complex, and one host-derived protein immunoglobulin [Ig] kappa light chain VLJ region; accession no.BAC01690 [4,17].

In the present study, using an indirect ELISA method, we have conducted a prospective study for demonstrating Ag 85 complex in sera from TB patients using a mAb against the complex. The data demonstrate that the positivities for Ag 85 complex antigen in cases of confirmed and clinically diagnosed TB patients were 96% (23/24) and 79% (82/104) respectively, while the positivity for patients in the non-tuberculosis group was 14% (10/69). The reason for these false positive results is unclear but latent infection cannot be ruled out. However, Ag 85 complex was not detected in any of the healthy control group. Overall, the indirect ELISA method yielded 82% sensitivity and 86% specificity for the diagnosis of TB using the mAb against Ag 85 complex.

There are not many reports about the detection of Ag 85 complex in sera from TB patients. Bentley-Hibbert et al. [25] measured the complex antigen in serum and urine by a mAb-based dot-immunobinding assay in 56 patients and controls with known skin test reactivity. The median serum Ag 85 complex levels were higher in patients with active TB than in patients with non-TB pulmonary disease or in healthy controls, suggesting that measurement of circulating Ag 85 complex might be developed into a diagnostic test for active tuberculosis infection. Similarly, Sánchez-Rodríguez et al. [26] determined the IgG antimycobacterial antibody response to Ag 85 complex antigen and showed a sensitivity of 72% and a specificity of 100%. Overall, these findings support our results, suggesting that detection of Ag85 complex might be developed into a diagnostic test for TB. However, one study has shown lower sensitivity with respect to examination of circulating Ag85 complex in patients with active TB [27].

Several previous studies have reported that Ag 85 complex is the immunodominant antigen in all mycobacteria including lepra bacillus and environmental mycobacteria, so it may give false results with other mycobacterial diseases [28]. However, when we evaluated the expression of Ag 85 complex in sera from twenty-five leprosy patients using the same protocol, the results were negative in all but one patient (unpublished observations). In any case, even though the lepra bacillus belongs to the same genus as the tuberculosis organism and has Ag 85 complex, it produces a clinical picture of leprosy that differs vastly from that of TB. Therefore, it is not difficult to rule out leprosy clinically as distinct from other infections. Moreover, the presence of Ag 85 complex in environmental mycobacteria and BCG would be unlikely to influence the interpretation of our results, because almost all the controls (i.e. non-TB patients and healthy subjects) included in the study were vaccinated with BCG and exposed to the same environment.

In this study, we have used an indirect ELISA method to detect *M. tuberculosis *Ag 85 complex in sera from TB and non-TB patients. Indirect ELISA is technically very simple and affordable in underdeveloped and developing countries. In the absence of sophisticated methods, such as molecular methods based on nucleic acid amplification and T cell-based immunological tests, indirect ELISA has become widely accepted for diagnosing tuberculosis and would potentially decrease the cost of the antibody reagent compared to the traditional sandwich ELISA system.

## Conclusion

Detection of Ag 85 complex in TB patient sera by indirect ELISA using a mAb to Ag 85 complex gives reliable diagnosis and does not give false results with other non-tuberculosis diseases. It could be used for developing an immunodiagnostic assay with increased sensitivity and specificity. Such a test would be rapid, sensitive and cost-effective and could be performed easily in any standard pathology laboratory.

## Competing interests

The author(s) declare that they have no competing interests.

## Authors' contributions

RSK carried out the study design, data collection, statistical analysis, data interpretation, literature search and manuscript preparation. ANR and SSR carried out the experiments and data collection. VSA and SSK collected the data from all patients. HJP participated in the preparation of the manuscript and data interpretation. GMT provided assistance in preparation of the manuscript, data interpretation, study design and collecting funds. HFD supervised the study design, statistical analysis, data interpretation, manuscript preparation and literature search. All authors read and approved the final version of the manuscript.

## Pre-publication history

The pre-publication history for this paper can be accessed here:


